# Comparison of the lower uterine segment in pregnant women with and without previous cesarean section in 3 T MRI

**DOI:** 10.1186/s12884-019-2314-7

**Published:** 2019-05-08

**Authors:** Janine Hoffmann, Marc Exner, Kristina Bremicker, Matthias Grothoff, Patrick Stumpp, Holger Stepan

**Affiliations:** 1 Department of Obstetrics, University of Leipzig, Liebigstrasse 20a, 04103 Leipzig, Germany; 2 Department of Radiology, University of Leipzig, Liebigstrasse 20, 04103 Leipzig, Germany; 3 Department of Radiology, University of Leipzig - Heart Center, Struempellstrasse 39, 04289 Leipzig, Germany

**Keywords:** Lower uterine segment, Previous cesarean section, Pregnancy, Diagnostic, MRI, Comparison to normal

## Abstract

**Background:**

Prenatal risk stratification of women with previous cesarean section (CS) by ultrasound thickness measurement of the lower uterine segment (LUS) is challenging. There is a wide range of proposed cutoff values and a valuable algorithm for selection before birth is not available. Using 3 T magnetic resonance imaging (MRI), we aimed to identify possible shortcomings of the current protocols used for birth selection after CS. Therefore, we evaluated anatomic and morphologic differences of the LUS and its thickness in patients with CS and those without. Possible impact factors on LUS thickness were studied.

**Methods:**

We retrospectively analyzed 3 T MRI scans of 164 pregnant women in their second or third trimester, with (patient group, *n* = 60) and without previous CS (control group, *n* = 104). Sagittal T2-weighted images were studied. Normal findings of the LUS in MRI, reliability of MRI measurements, as well as factors influencing LUS thickness were assessed. MRI findings were compared to intraoperative findings.

**Results:**

MRI provided good intra- (ICC 0.872) and fair inter-rater reliability (ICC 0.643). The relationship of the LUS and the cesarean scar to the surrounding anatomical structures and also its morphology varied strongly in patients and controls. Scar identification was possible in only 9/60 (15.0%) patients. The LUS was thinner in patients (1.9 ± 0.7 mm) than in controls (2.7 ± 1.3 mm). An LUS thinning up to 1 mm was observed in 23% of women without a previous CS and in 34% of women with normal intraoperative findings. Suspicion of a uterine dehiscence (LUS thickness < 1 mm) was only found in the patient group (5/59 (8.5%)) and was intraoperatively confirmed. In controls, LUS thickness was influenced by fetal weight, gestational age and amniotic fluid amounts.

**Conclusion:**

Variability in anatomy, thickness and morphology seem to limit common prenatal LUS imaging diagnostics. Therefore, we consider that diagnostic protocols must be re-evaluated and imaging should be adjusted to the individual patient conditions. Due to its independency of ultrasound limitations, an additional MRI might be useful for altered anatomy and impaired ultrasound conditions. An LUS thinning up to 1 mm might be a normal finding and should be further investigated as reference value.

## Background

With high success rates of up to 87%, vaginal birth (VBAC) is frequently offered to women after previous cesarean section (CS) [1, 2]. A rare complication is uterine rupture during birth. Despite its low incidence of 0.4–0.9% but in consideration of its devastating outcome, risk stratification by additional prenatal ultrasound diagnostics of the lower uterine segment (LUS) has been a hot topic for the last 20 years [3–5]. Today, a correlation between LUS thickness and the risk for uterine rupture can be assumed, but neither useful reference values nor even the benefit of prenatal LUS thickness measurement have been clearly demonstrated [4, 6]. Various studies have investigated different measurement approaches using 2D- and 3D-techniques in transvaginal and transabdominal ultrasound [1, 5, 7–9]. The inconsistent results with wide-ranging reference values for the very thin structure of the LUS and experience from clinical routine raise reasonable doubts about the usefulness of LUS thickness measurement for birth selection in clinical practice [10]. Nevertheless, ultrasound is the first-line noninvasive imaging technique and allows an overview of morphology and dimensions of the scarred uterus after CS [6]. We hypothesize that the current protocols are insufficient but might be improved if specific characteristics of the LUS would be considered in diagnostics.

As a complementary and safe noninvasive imaging modality during pregnancy, MRI is a highly interesting approach but not yet sufficiently investigated [11–14]. With our recent scientific results we demonstrated MRI to be an adequate additional noninvasive image modality for prenatal LUS diagnostics in patients with previous CS. Because of the different technique and the associated advantages and disadvantages MRI might be a useful additive diagnostic tool when ultrasound conditions are limited [15, 16]. We furthermore believe that MRI as a different imaging approach without the requirement of an adequate acoustic window also holds the potential of providing new insights and offers the possibility of finding a more reliable diagnostic algorithm. A comparison of LUS findings and thickness between pregnant women with and without previous CS in MRI has never been done before. To improve the distinction of pathologic and normal findings, we sought to study whether there are specific morphologic signs in patients with a scarred uterus compared to those without a scar which might be useful for future risk stratification. Because an LUS thinning is normal merely by the growing pregnancy we compared the LUS thickness of patients with and without CS. Thereby we aimed to study if thickness measurement by MRI is possible to differentiate a pathologic uterine thinning due to a uterine dehiscence from a ‘normal’ pregnancy related LUS thinning.

## Methods

We retrospectively studied 164 patients who underwent pelvic MRI during pregnancy in our prenatal care center at the University of Leipzig. Three twin pregnancies were included for analysis of LUS anatomy and morphology, but due to assumed altered intrauterine pressure, they were excluded from analyses regarding LUS thickness. Indications for MRI were fetal anomalies (*n* = 75), abdominal pain in patients with (*n* = 5) or without previous CS (*n* = 5), a suspected uterine dehiscence in ultrasound after previous CS (*n* = 11), previous CS with normal ultrasound findings (*n* = 25) and birth planning for breech presentation (*n* = 43). We defined a patient group with previous CS and a control group without history of CS. Patient characteristic was obtained from the electronic medical records in the ViewPoint and SAP 710 documentation systems.

All MRI examinations were performed on a 3 T MRI system (MAGNETOM Trio, Siemens Healthcare, Erlangen, Germany) with a two-channel body matrix coil. Only T2-weighted sequences in a sagittal slice orientation with diagnostic image quality were included in the analysis. Diagnostic image quality was agreed if the whole lower uterine front wall, the LUS and the cervix were recorded including the sidewalls, if the cervical channel was completely visible in at least one slice and if the urinary bladder was directly juxtaposed to the front of the uterus.

Two different MRI sequences, a Half-Fourier Acquired Single-shot Turbo spin-Echo (HASTE) and a True Fast Imaging with Steady-state Precession (TrueFISP) sequence, with a similar average in-plane resolution were used. The detailed MRI-protocol is given in Table 1.

**Table 1:** MRI-protocol of the included sagittal T2-weighted sequences

Sequence	ST^c^ (mm)	TR^d^ (ms)	TE^e^ (ms)	Matrix (mm)	In-plane Resolution (mm)	n
T2-HASTE^a^	4	1500	95	320 × 242	0.94 × 1.24	75
T2-TRUFI^b^	4	6.62	2.66	320 × 256	0.87 × 1.09	89

^a^T2-HASTE (T2-weighted Half-Fourier Acquired Single-shot Turbo spin-Echo sequence), ^b^ T2-TRUFI (T2-True Fast Imaging With Steady-state Precession sequence), ^c^ ST (slice thickness), ^d^ TR (Time to Repeat), ^e^ TE (Time to Echo)

**Fig. 1:**
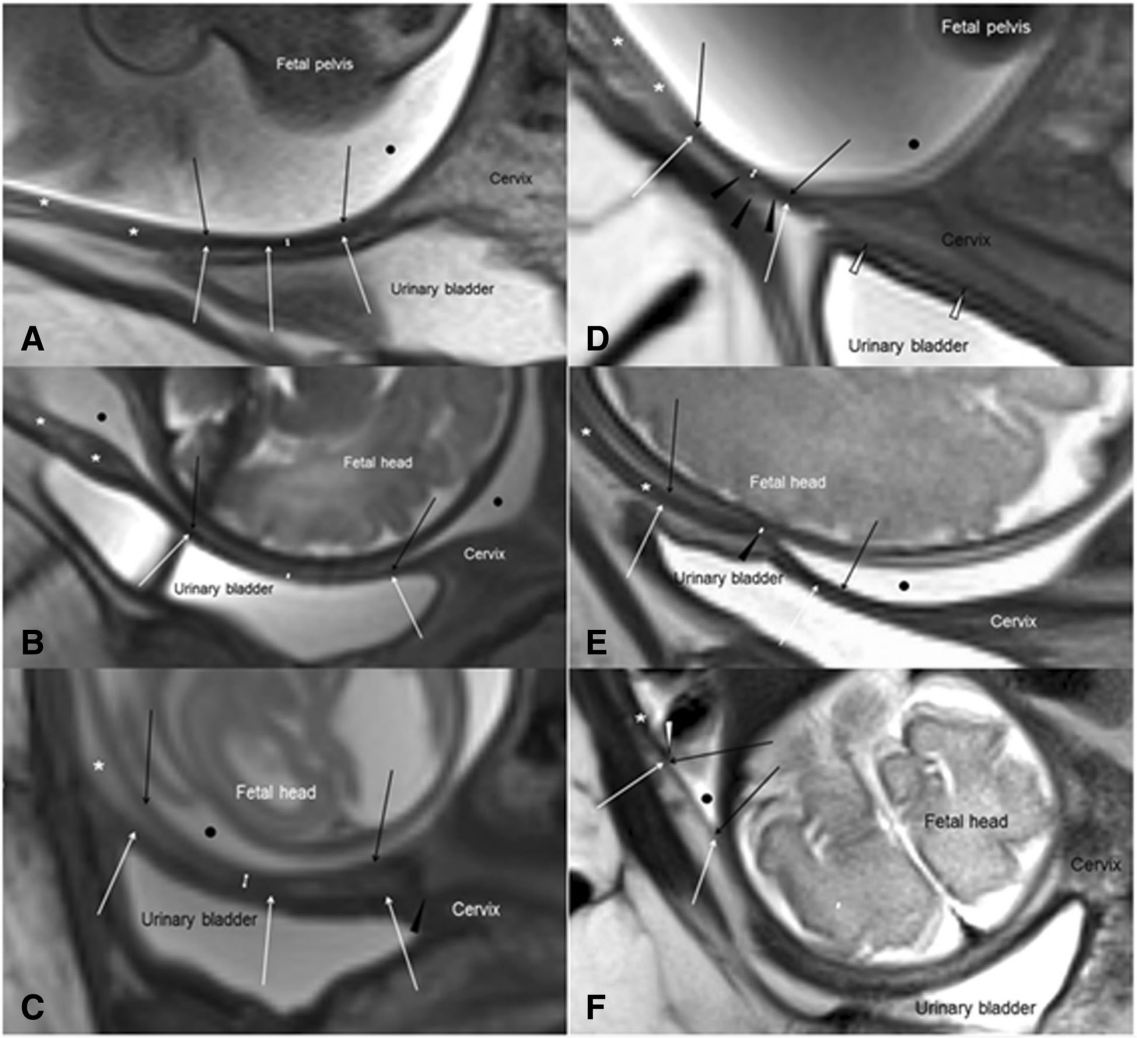
Thickness measurement of the lower uterine segment (LUS) and definitions in 3 T MRI. Findings also demonstrate possible pitfalls in ultrasound diagnostics. Black arrow = inner border of the LUS, white arrow = outer border of the LUS, white double headed arrow = measured LUS-thickness, asterisk = lower uterine front wall, black point = amniotic fluid. **a** Myometrial LUS thickness measurement from the interface amniotic fluid/ LUS (black arrows) to the interface urinary bladder wall/LUS (white arrows) when LUS and urinary bladder are well definable. **b** Full LUS thickness measurement from the interface amniotic fluid/LUS (black arrows) to the interface urinary bladder wall/urine (white arrows), an alternative in cases with undefinable interface urinary bladder wall/ LUS. **c** Measurements for atypically located hysterotomy scars within the cervix (black arrow head). Measurements in typical location (thin arrows) might miss the correct diagnosis. **d** Demonstration of a hypointense LUS with characteristics of a uterine scar (black arrow heads) in an atypical location above the urinary bladder. Due to altered anatomy, common measurement standards would not be appropriate. **e** Illustration of an important limitation of full LUS thickness measurement. Due to the varying thickness of the urinary bladder wall and the interstitial tissue, this approach would overestimate the “real” LUS thickness up to several millimeters that had to be measured at the cesarean scar level (black arrow head). **f** Typical finding of a uterine dehiscence (abrupt interruption of the LUS/myometrium with change in diameter (white arrow head) and a lamellar-like thinning (thin arrows)). The thin dehiscent uterine front wall is often not definable from adjacent fetal head or urinary bladder wall and might be missed if LUS thickness was only measured in the typical location at the urinary bladder level

**Fig. 2:**
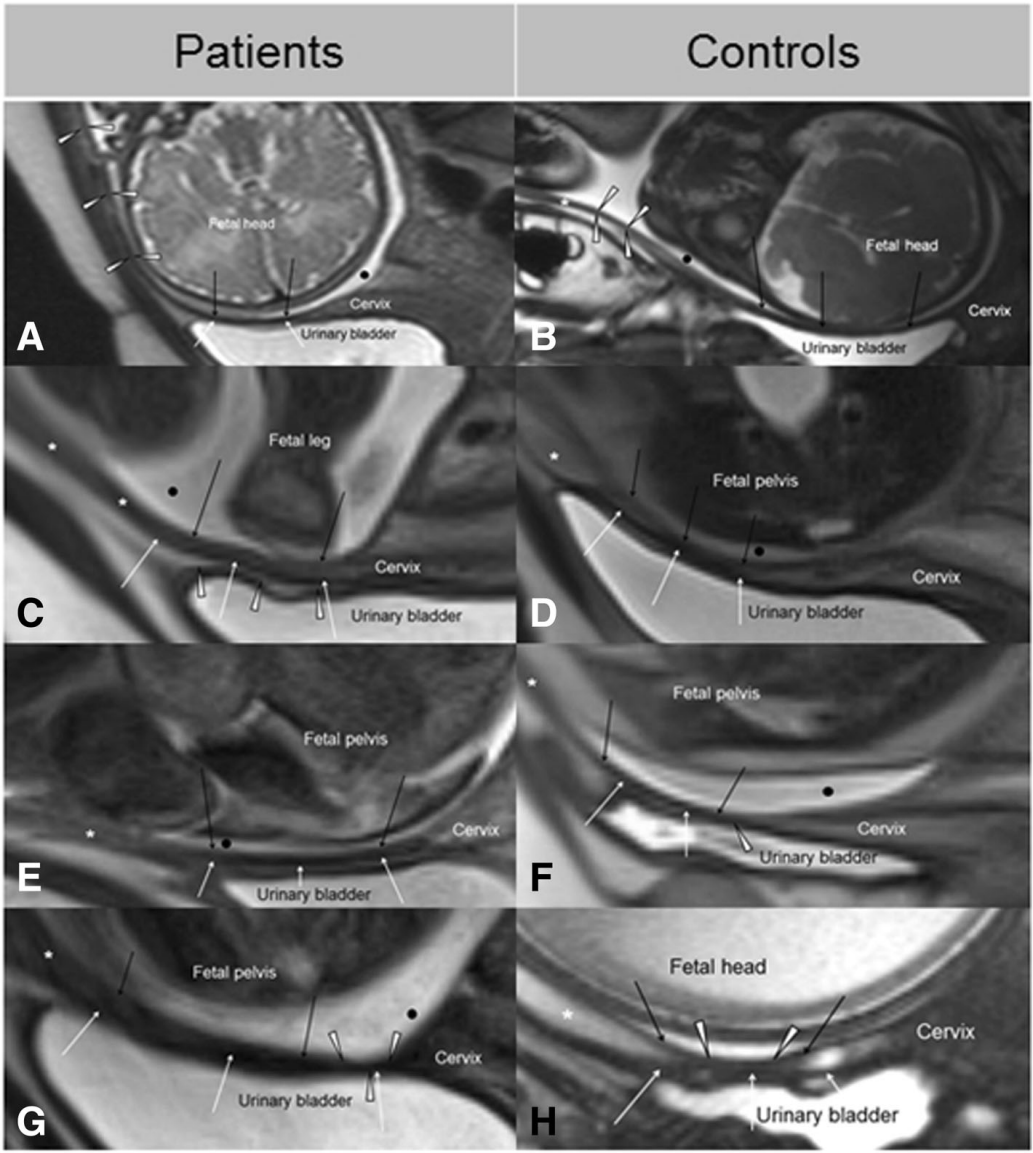
Findings in patients with cesarean section (CS) on the left and in controls without CS on the right. By comparing the images, difficulties in LUS diagnosis and in finding valuable reference values for LUS thickness become clearer. Black point = amniotic fluid, black arrows = inner border of the LUS, white arrows = outer border of the LUS, asterisk = lower uterine front wall, black point = amniotic fluid. As demonstrated in comparison (**a**) versus (**b**), thinning of the LUS (thin arrows) and/or the lower uterine front wall (white arrow heads), as well as unclear definitions of LUS and bladder wall occurs in both scarred and unscarred uteri. In contrast, comparison of (**c**) versus (**d**) shows that a clear definition of a well-developed LUS from the urinary bladder wall is also commonly found after CS (white arrow heads). Comparison of (**e**) versus (**f**) shows that the MRI-signal of the LUS can be hypointense in women with and without previous CS. Even local thinning with an abrupt change of LUS diameter, which is assumed to be the most specific characteristic after CS (**g**), can probably related to myometrial contractions, occasionally also be observed in women without a uterine scar (**f**) and (**h**), (white arrow heads)

Three independent experienced investigators (J.H., M.E. and K.B.) performed off-line image analysis on a separate workstation (Syngo.Plaza, Siemens Healthcare, Erlangen, Germany). Anatomic (LUS location mainly (> 80%) behind or above the urinary bladder, measurement point location behind or cranial to the bladder) and morphologic criteria of the LUS (hypointense or isointense signal intensity to the adjacent tissue, differentiability to the urinary bladder wall, present or absent signs indicating a scar) were described and compared between patients and controls (Figs. 1, 2).

Blinded to of the patient history and previous findings or measurements, LUS thickness was measured once by 3 investigators to obtain inter-rater reliability (J.H., M.E., K.B.) and twice by one investigator (J.H.) to obtain intra-rater reliability. Measurements were performed at the thinnest area of the LUS or, if visible, at the scar. Signs indicating the hysterotomy scar were irregular or blurred boundaries, a circumscribed local thinning, prompt change of diameter and/or texture inhomogeneity (Fig. 1c-e). If it was identifiable, the myometrium LUS was measured by putting one cursor on the interface of amniotic fluid/LUS and the second cursor on the interface of urinary bladder wall/LUS (Fig. 1a) or on the outer LUS borderline (Fig. 1d, e), respectively. Full LUS thickness was determined alternatively by putting the second cursor on the interface urine/urinary bladder wall, if the LUS was not clearly definable (Fig. 1b). LUS thickness was classified as normal (>2 mm), slightly (1.5.-2.0 mm), moderately (1.0–1.5 mm) or severely thinned (<1.0 mm). A uterine dehiscence was diagnosed if the LUS was severely thinned and appeared lamellar-like (Fig. 1f). LUS thickness was compared between patients and controls.

Several preconditions were analyzed regarding their influence on LUS thickness. These were fetal presentation (head, breech or transverse/oblique), estimated amounts of amniotic fluid between the fetus and the inner LUS borderline (little (maximum a thin liquid layer) or large amounts), filling level of the urinary bladder (sufficient (bladder full-filled/urinary fold extended) or insufficient (maximum partly filled/urinary fold not extended)), gestational age, estimated fetal weight from a near-term (5 ± 7 days) ultrasound biometry and number of previous CS.

If available, MRI findings were compared to intraoperative findings, obtained from the electronically stored operation report.

For statistical analyses, IBM SPSS Statistics 22 was used. As derived from Kolmogorov-Smirnov and Levene’s test, most parameters were normally distributed and equal in variance; hence, parametric tests were used. Descriptive statistics were performed. Inter- and intra-rater reliability for LUS thickness measurements were determined, using intra-class-correlation (ICC) analysis with a two-way mixed model for metric data and Kappa values for categorical data. Agreement was considered fair to good if the ICC or Kappa value were 0.45–0.75, good if 0.75–0.90 and excellent if > 0.90. To identify influencing factors on LUS thickness, linear Pearson correlation, Chi^2^- tests or ANOVA with post hoc analyses using Bonferroni correction were conducted. Independent influencing factors on LUS thickness were evaluated using linear or bivariate regression analyses. *P*-values of 0.05 were considered statistically significant. The 95% confidence intervals are given.

## Results

Patient characteristics are shown in Table 2. We found 95 (57.9%) fetuses or the leading fetus in head presentation, 67 (40.9%) in breech and 2 (1.2%) in transverse/oblique presentation. The mean estimated fetal weight was 2008 ± 900 g.

**Table 2:** Patient characteristics of the total study group (*n* = 164). Parameters are given as the mean ± standard deviation or absolute (percentage) amount. CS = cesarean section

	All patients	Prior CS	No CS	P (prior /no CS)
Maternal age at examination (years)	30.6 ± 5.3	32.4 ± 4.8	29.9 ± 4.7	0.001
Gestational age at MRI-examination (weeks)	32.4 ± 5.4	32.0 ± 5.5	33.1 ± 5.1	0.208
Parity				< 0.001
- Nulliparous	79 (48.2%)	–	79 (75%)	
- one previous delivery	56 (34.1%)	40 (67%)	16 (16%)	
- two previous deliveries	17 (10.4%)	12 (20%)	5 (5%)	
- > two previous deliveries	12 (7.3%)	8 (13%)	4 (4%)	
Patients without prior cesarean section	104 (63.4%)	–	–	
Patients with prior cesarean section	60 (36.6%)	–	–	
- One previous cesarean sectio	50 (83.4%)			
- Two previous cesarean sections	5 (8.3%)			
- > two previous cesarean sections	5 (8.3%)			

In 75 (45.7%) patients, a T2-weighted HASTE sequence was used, and in 89 (54.3%) patients a T2-weighted TrueFISP sequence was used. Image quality was good to excellent in 147 (89.6%), and only slightly or moderately limited but diagnostically appropriate in 17 (10.4%) patients. Intra-rater reliability of LUS thickness measurement was good (ICC 0.872, *p* < 0.001) and inter-rater reliability was fair (ICC 0.643, p < 0.001).

Anatomy and morphology of the LUS were heterogeneous, irrespective of whether women had a previous CS or not (Figs. 1, 2). Mostly, the LUS (151/164 (92%)) and accordingly, also the point of measurement (146/151, 97%) were determined behind the urinary bladder. In 5/151 (3%) patients, the point of measurement was above the urinary fold. In a minority of cases (13/164 (8%)), both the LUS and the point of measurement were situated above the bladder (Fig. 1d, f).

**Fig. 3:**
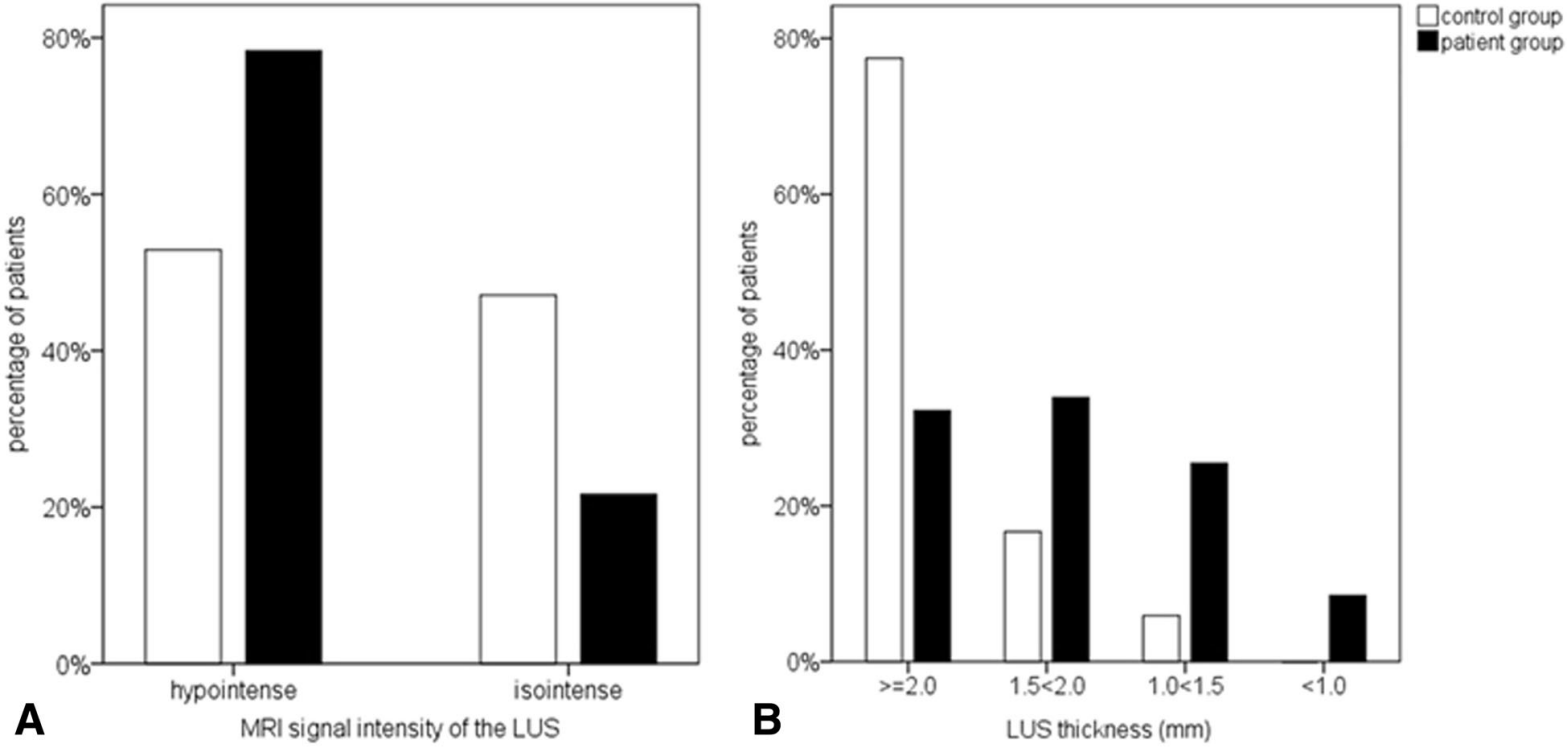
Incidence of hypointense and isointense MRI signal of the LUS, referenced to the adjacent myometrium (**a**) and degree of LUS thinning (**b**) in patients with cesarean section (black bars) and controls with unscarred uteri (white bars). LUS thickness ≥ 2.0 mm was defined as normal; 1.5 < 2.0 mm slightly; 1.0 < 1.5 mm moderately; and < 1.0 mm severely thinned or suspected uterine dehiscence

As demonstrated in Fig. 2, the comparison of patients and controls did not provide specific morphologic characteristics of uterine scar tissue. Clear definition of the interface LUS/urinary bladder wall as demonstrated in Fig. 1a was possible in 84/146 (58%) cases, without significant differences between controls and patients (*p* = 0.234). Thus, the full LUS thickness as shown in Fig. 1b was alternatively determined in 62/146 (42%) cases. In comparison to the adjacent myometrium, the LUS appeared more often hypointense in the patient group (*p* = 0.001) but also frequently in the controls (Fig. 2e, f, and Fig. 3). The inter-rater reliability for scar detection by the described MRI characteristics ranged widely (8–28/60 (13–47%)). Only in 9/60 (15.0%) patients with CS the uterine scar was consistently described by all observers (Fig. 1c, e), and in 5/104 (4.8%) controls, uterine scar signs were even falsely described (Fig. 2f, h). In 4/60 (6.7%) patients, the uterine scar was depicted within the cervical myometrium (Fig. 1c). The absence of a uterine scar was more consistently diagnosed in the controls (96/104 (93.0%)).

In patients, the LUS was thinner than that in controls and was unaffected by the number of previous CS (Table 3). A slight to moderate LUS thinning was more frequent in patients (*n* = 37 (62%)) but also occurred in controls (*n* = 24 (23%), *p* < 0.001) (Fig. 3b). In contrast, a severe LUS thinning and suspicion of uterine dehiscence were only observed in the patient group (5/59 (8.5%)) (Fig. 1f, and Fig. 3b). These findings remained significant, even if those patients were excluded who obtained MRI because of severe LUS thinning or uterine dehiscence in ultrasound.

**Table 3:** Differences of the LUS (lower uterine segment) thickness related to possible influencing factors in the total study group with singleton pregnancies (*n* = 161). Significance levels (p), obtained from ANOVA analysis or Chi^2^ tests are given. CS = cesarean section *p < 0.05 in comparison to the group < 24 + 0 gw, **p < 0.05 in comparison to the group 24 + 0–27 + 6 gw, ***p < 0.05 in comparison to the group 2000 < 3000 g

	Mean LUS-thickness (mm)			p
Parity				0.320
- Nulliparous	2.7 ± 1.3			
- one previous delivery	2.2 ± 1.1			
- two previous deliveries	2.0 ± 0.9			
- > two previous deliveries	2.3 ± 1.0			
Previous cesarean section				< 0.001
- yes	1.9 ± 0.7			
- no	2.7 ± 1.3			
Number of previous cesarean section				0.763
- one	1.9 ± 0.7			
- two	2.1 ± 1.0			
- >two	1.8 ± 0.6			
	All patients	Prior CS	No CS	P (prior/no CS)
Gestational age at MRI-examination				
- < 24 + 0	3.1 ± 1.7	2.3 ± 1.0	4.1 ± 1.9	0.041
- 24 + 0–27 + 6	3.2 ± 1.9	2.0 ± 0.8	3.8 ± 2.1	0.018
- 28 + 0–31 + 6	2.5 ± 0.8	1.9 ± 0.6	2.8 ± 0.7	0.002
- 32 + 0–35 + 6	2.2 ± 0.8**	1.7 ± 0.6	2.5 ± 0.7	0.001
- 36 + 0–42 + 0	2.1 ± 0.8*,**	1.8 ± 0.8	2.2 ± 0.7	0.089
Estimated fetal weight (g) at examination				
- <1000 g	3.0 ± 1.4***	2.0 ± 0.8	3.6 ± 1.3	0.001
- 1000 < 2000 g	2.8 ± 1.8***	1.7 ± 0.5	3.3 ± 2.0	0.023
- 2000 < 3000 g	1.9 ± 0.8	1.6 ± 0.5	2.3 ± 0.8	<0.001
- ≥3000 g	2.2 ± 0.9	2.0 ± 1.0	2.4 ± 0.7	0.309
Fetal position				
- Head presentation	2.4 ± 1.4	1.8 ± 0.7	3.0 ± 1.6	<0.001
- Breech presentation	2.5 ± 0.9	2.2 ± 0.8	2.5 ± 0.8	0.240
- Transvers/oblique presentation	2.1 ± 1.1	1.3	2.9	
Amounts of amniotic fluid between fetus and LUS				
- none-little amounts	2.3 ± 0.8	1.8 ± 0.6	2.5 ± 0.9	<0.001
- larger amounts	2.7 ± 1.6	1.9 ± 0.9	3.3 ± 1.7	<0.001
Filling level of the urinary bladder				
- sufficient	2.4 ± 1.2	1.9 ± 0.6	2.8 ± 1.4	<0.001
- insufficient	2.5 ± 1.2	1.9 ± 1.0	2.6 ± 1.0	0.003

**Fig. 4:**
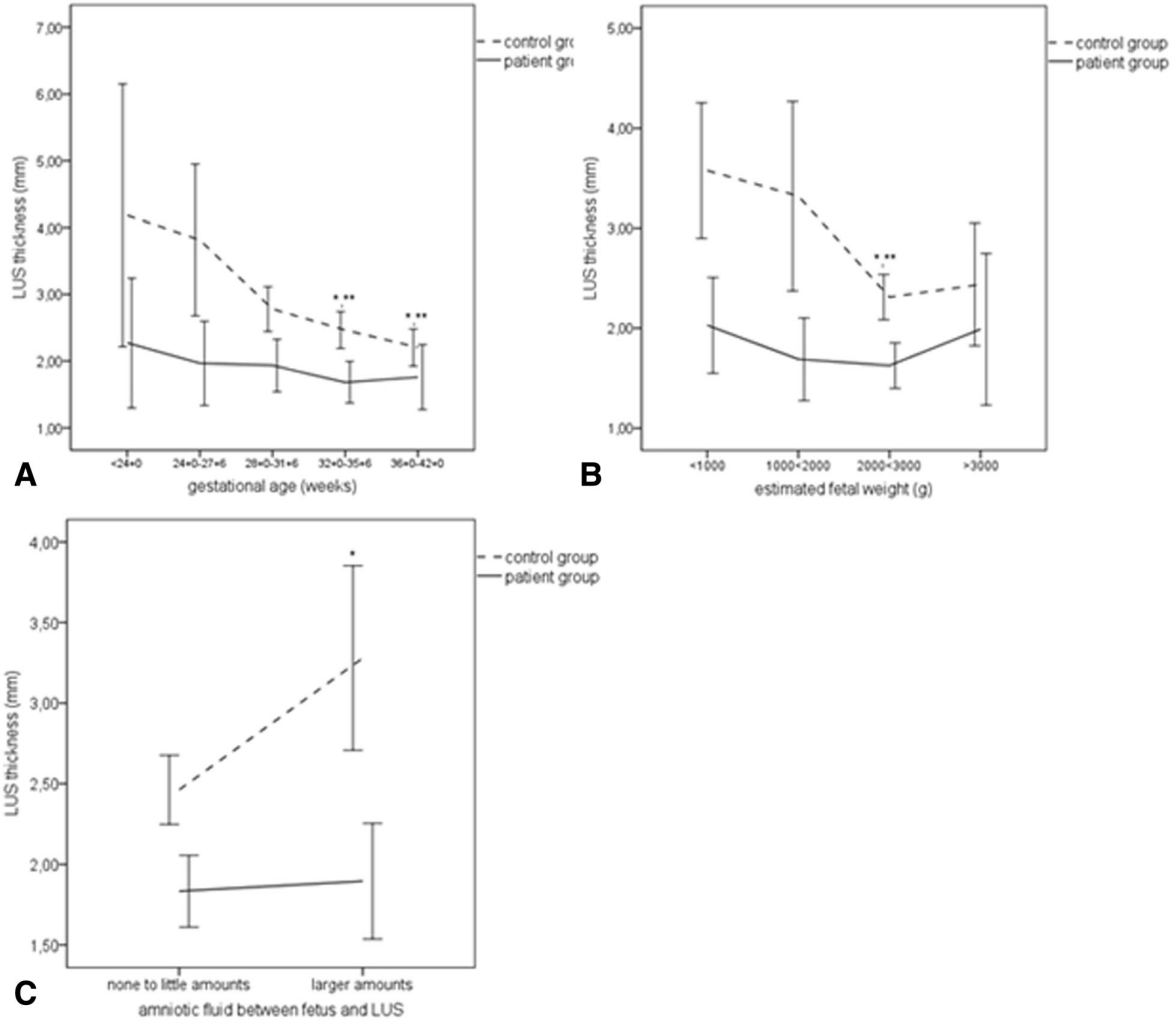
Differences of the LUS in relation to factors associated with intrauterine pressure such as gestational age (**a**), estimated fetal weight (**b**) and estimated amounts of amniotic fluid between fetus and LUS (**c**) in controls without cesarean section (broken lines) and patients (continuous lines). Error bars represent the 95% confidence interval of each group. **a**: *p < 0.05 compared to group <24 + 0 gw, **p < 0.05 compared to group 24 + 0–27 + 6 gw (**b**): *p < 0.05 compared to group <1000 g, **p < 0.05 in comparison to the group 1000 < 2000 g (**c**): *p < 0.05

Tables 3 and 4 demonstrate fetal weight, gestational age and the amount of amniotic fluid between the fetus and the inner LUS borderline as significant influencing factors on LUS thickness in all patients and in patients with and without previous CS. Estimated fetal weight (r = − 0.263, *p* = 0.001) and gestational age (r = − 0.313, p < 0.001) showed slightly negative linear correlations with the LUS thickness. The LUS thickness differed significantly between the groups with estimated fetal weight < 1000 g vs. 2000 < 3000 g (*p* = 0.002) and between the second and third gestational trimester (< 32 + 0 weeks MW 2.8 ± 1.5 vs. ≥32 + 0 weeks, MW 2.1 ± 0.8, *p* < 0.002) (Table 3). The influence of all factors was only significant in the control but not in the patient group (Table 5, Fig. 4).

**Table 4:** Univariate and multivariate regression analyses regarding possible influencing factors on lower uterine segment thickness in the total study group with singleton pregnancies (*n* = 161). The results are presented with *p*-value, Beta coefficient and its 95% confidence interval [95% CI]. *p < 0.05, **p < 0.001

	Univariate regression analysis	Multivariate regression analysis
Previous cesarean section	<0.001; − 0.362 [− 1.238-(− 0.526)]**	<0.001; − 0.444 [− 1.466-(− 0.760)]**
Gestational age (weeks)	<0.001; − 0.315 [−0.102-(−0.037)]**	<0.001; −0.670 [−0.227-(−0.080)]**
Estimated fetal weight	0.003; − 0.242 [− 0.001–0.000]*	0.036; 0.344 [0.00–0.001]*
Fluid between fetus and LUS	0.022; 0.180 [0.062–0.808]*	0.148; 0.107 [− 0.098–0.640]

**Table 5:** Multivariate regression analyses in the patient (*n* = 59) and control groups (*n* = 102) with singleton pregnancies regarding possible influencing factors on lower uterine segment thickness. The results are presented with *p*-value, Beta coefficient and its 95% confidence interval [95% CI]. *p < 0.05, **p < 0.001

	Multivariate regression analysis Patient group	Multivariate regression analysis Control group
Gestational age (weeks)	0.343; − 0.130 [− 0.052–0.018]	<0.001; − 0.733 [− 0.280-(− 0.090)]**
Estimated fetal weight	0.615; 0.189 [0.000–0.001]	0.088; 0.324 [0.000–0.001]
Fluid between fetus and LUS	0.530; 0.096 [0.565-(− 0.294)]	0.017; 0.220 [0.113–1.139]*

Intraoperative findings were available in 73 cases and because of a vaginal delivery not available in 78 cases. In 13 cases, women did not deliver in our clinic and outcome was therefore not available. 36/60 patients with previous CS had a repeated CS because of patients demand (*n* = 11), > 1 previous CS (*n* = 2), previous uterine rupture (*n* = 3), suspected uterine dehiscence (n = 11), fetal diseases (*n* = 6), pathologic CTG or stucked labour (n = 2) and breech presentation (n = 1).

In MRI, a clinically dehiscent LUS (*n* = 5) was described at least as a moderate LUS thinning (n = 3) and as a severe thinning/dehiscence in two cases. An intraoperatively remarkably thin LUS was described in MRI as a slight (*n* = 9) or a moderate (n = 3) thinning. An intraoperatively not remarkably thin LUS (*n* = 59) was also normal in MRI in 39 cases and appeared slightly thinned out in 13 (22%) and moderately thinned in 7 (12%) cases (total *n* = 20, 34%).

## Discussion

Our results show that 3 T-MRI is a valuable additional tool for noninvasive LUS diagnostics [15]. With fast T2-weighted sequences, relevant uterine structures and particularly the very thin LUS can be reliably visualized, independent of ultrasound limitations. Due to the higher field strength of 3 T, spatial resolution and tissue contrasts were better in our study than those in previous studies using 1.5 T [11, 12]. Consistent with previous studies, our data indicate that urinary bladder filling level does not influence LUS thickness, and thus, a partial filling level might be preferred for better patient comfort [8].

We found various factors that might explain the difficulties of ultrasound LUS diagnostics and the wide range of ultrasound-derived reference values for prenatal selection of patients with previous CS. The consideration of these factors might help to improve the value of current prenatal screening with ultrasound when VBAC is planned.

Primarily, as previously demonstrated the LUS morphology and the scar localization are highly variable [16]. Although it is appropriate to measure behind the bladder in most cases, due to altered anatomy or atypical incision at previous CS, in some cases LUS measurements have to be performed cranial to the urinary bladder, within the lower uterine front wall or the cervical myometrium. Therefore, the combination of transabdominal and transvaginal ultrasound measurements, as already recommended by some authors, is mandatory. A further diagnostic gap might be closed by an additional standard examination of the lower uterine front wall [4, 17–20]. We also consider an additional MRI to be useful if ultrasound conditions are limited and anatomy is altered.

Furthermore, our study demonstrates that full LUS thickness measurement bears the risk of becoming distorted by the bladder wall or the individually varying thickness of the interstitial layer between bladder and LUS. Due to good standardization, this measurement may provide a better reproducibility, but largely independent of LUS location and surrounding structures, we consider the myometrial LUS thickness to be the more promising parameter [6, 19, 21]. Full LUS thickness measurement should be determined alternatively if the LUS/ myometrium is not definable. Difficulties in defining the LUS/bladder interface are common in MRI and in ultrasound [6, 21]. Since this finding is frequently seen in both patients and controls, it should not be misinterpreted as adhesion or scar after CS.

Difficulties in LUS thickness measurement are aggravated since the uterine scar and therefore the correct point of measurement cannot reliably be determined with ultrasound or MRI [22]. In fact, LUS morphology was strongly heterogeneous in MRI, irrespective of a previous CS. Except in the cases of a severe (LUS thickness < 1 mm) or lamellar-like LUS thinning indicating a uterine dehiscence, we did not find specific tissue characteristics reliably indicating the hysterotomy scar. A lower detection rate of the uterine scar in this (15%) and our previous study (44%) can be explained by the different study design. Whereas the history of CS was unknown to the observers in this study, only patients with one previous CS were included into the previous study so that history of CS was known.

The observed limitations in imaging and measurement are certainly one of the main reasons why there is still no reliable reference value for LUS thickness allowing pre-labor risk selection after CS. Moreover, the comparison of women with and without previous CS showed that LUS thinning in the course of pregnancy is normal and related to increasing intrauterine pressure with advancing pregnancy. LUS thinning down to 1 mm was found in a remarkable number of pregnancies of the control group and must be considered to be normal [22, 23]. This hypothesis is substantiated since a high number of patients with a slight to moderate thinning down to 1 mm in MRI had normal intraoperative findings. Hence, the risks for uterine defects are probably overestimated with reference values of ≥2 mm for LUS thickness, and only a uterine dehiscence bears a risk for a uterine rupture [4, 19]. Although not sufficiently studied in the literature, there is one older ultrasound study supporting our results [22]. Comparing women with scarred and unscarred uterus, the authors also concluded that an LUS thickness > 1 mm might be normal. In the latest multicenter study, uterine defects were more frequently observed in patients with full LUS thickness < 2 mm (mean 1.6 ± 0.3 mm) [4]. As the mere myometrial LUS thickness must be considered to be significantly lower, these results also strengthen our findings. Because a uterine dehiscence was correctly diagnosed with a reference value of 1.0 mm for LUS thickness we think that further investigation should be focused on its usefulness and safety for pre-labor selection after CS.

Vulnerability of the LUS is certainly not only related to thickness but also to tissue properties. Recent studies have demonstrated that increased connective and decreased myometrial tissue result in decreased elasticity and contractility after CS [24, 25]. This finding might explain the absence of influencing factors on LUS thickness and the lower variability of LUS thickness in the patient group due to increased stiffness of the tissue. However, the abovementioned histological alterations cannot be depicted using T2-weighted sequences. Because of the strong individual heterogeneity of LUS morphology, we furthermore do not think that T2-weighted MRI or ultrasound-derived tissue characteristics are appropriate for risk stratification as previously suggested [12, 13]. As suggested by small feasibility studies ultrasound-elastography or advanced MRI sequences such as diffusion tensor imaging (DTI) might be more suitable for scar tissue characterization after CS [26, 27].

The main limitation of our study is its retrospective design. Despite careful patient selection, different indications and MRI sequences potentially compromise study power. Comparisons to a concomitant ultrasound examination or longitudinal measurements were not possible. Due to the study design it was not possible to evaluate useful reference values or an appropriate selection protocol.

## Conclusions

This largest study so far investigating and comparing MRI findings of the LUS in patients with and without previous CS indicates necessity of a new diagnostic algorithm for prenatal LUS diagnostic. We think that this, similar to our model, should offer an adaption to the individual patient conditions. An additional MRI might be helpful in case of limited ultrasound conditions. Usefulness and safety of 1 mm as reference value should further be evaluated for risk selection in birth planning after CS.

## Abbreviations

CS: Cesarean Section
HASTE: Half-Fourier Acquired Single-shot Turbo spin-Echo sequence
ICC: Intraclass Correlation Coefficient
LUS: Lower Uterine Segment
MRI: Magnetic Resonance Imaging
MW: mean value
TrueFISP: True Fast Imaging with Steady-state Precession sequence
VBAC: Vaginal Birth After Cesarean Section
